# Broccoli byproduct-wheat straw silage as a feed resource for fattening lambs^1^

**DOI:** 10.1093/tas/txaa078

**Published:** 2020-07-20

**Authors:** Edris Partovi, Yousef Rouzbehan, Hasan Fazaeli, Javad Rezaei

**Affiliations:** 1 Department of Animal Science, Faculty of Agriculture, Tarbiat Modares University, Tehran, Iran; 2 Animal Science Research Institute of Iran, Agricultural Research, Education and Extension Organization, Karaj, Iran

**Keywords:** broccoli byproduct, lamb, lamb growth, protozoa, rumen

## Abstract

The effect of feeding broccoli byproduct-wheat straw silage [BBWS; 69:31 ratio, dry matter (DM) basis] on performance, microbial N synthesis (MNS), rumen, and blood parameters in Fashandy lambs were evaluated. Three diets, with equal metabolizable energy and crude protein (CP) with a forage to concentrate ratio of 27:73 (DM basis), were formulated in which forage (lucerne and wheat straw) was replaced by BBWS (0, 100, or 200 g/kg of diet DM). These were assigned to three groups (*n* = 15/group) in a completely randomized block design for a 70-d period in which diets were offered as a total mixed ration. For each animal, dry matter intake (DMI), average daily gain (ADG), in vivo apparent digestibility, MNS, N retention, rumen, and blood parameters were measured. The BBWS diets had no influence on DMI, ADG, feed conversion efficiency, in vivo apparent digestibility coefficients of DM, organic matter, CP, and ash-free neutral detergent fiber. Neither MNS and N retention nor serum concentrations of glucose, triglycerides, creatinine, cholesterol, urea N, triiodothyronine, thyroxine, total protein, albumin, and globulin were affected. Rumen pH, NH_3_-N, short-chain fatty acid concentrations, the ratio of acetic to propionic acid, and protozoa numbers were, also, not influenced. In summary, BBWS may be fed to Fashandy lambs up to 200 g/kg of diet DM without any adverse impacts on growth performance.

## INTRODUCTION

The increasing frequency and intensity of droughts in the world have led to shortages of common livestock feedstuffs (Alipour and Rouzbehan, 2010). Alternative feed sources, including agricultural waste byproducts, need to be investigated as ingredients for livestock diets. Broccoli (*Brassica oleracea* L. var. *italica*) byproduct comprised of stem and leaves could be an alternative.

The broccoli flower bud is harvested for human consumption and more than two-thirds of the plant (outer leaves and stems) is left in the field. The use of this discarded crop waste is a possible way to supply animal feedstuffs and decrease the environmental pollution. Globally, 20 million tonnes of cauliflower and broccoli crops are produced yearly and 15 million tonnes of byproduct are left in the field (Jian et al., 2017). In Iran, the estimated production of cauliflower and broccoli for the year 2016 was 60,000 tonnes with 45,000 tonnes of byproduct available for ruminant feed (FAO, 2018). Vegetable growers and ruminant farmers exist side by side in many parts of the world, and this could facilitate the use of these waste products as ruminant feed. However, the fast rate of spoilage due to high moisture (>70%) make the use of these waste products not practical, so ensiling is potentially a good preservation technique for broccoli byproduct (BB) to reach the ideal moisture content for ensiling is between 60% and 70% (McDonald et al., 1991). The preservation of the forage crops as silage is based on a fermentation process that lowers the pH and preserves the nutritive value of the fresh crop. The main principle is the production of lactic acid by the lactic acid bacteria from the metabolism of the water-soluble carbohydrates in the fresh crop. Since fresh BB contains about 186 g/kg dry matter (DM) soluble sugars (Wadhwa et al., 2006), mixing BB with dry feeds, such as straw, before ensiling probably results in good preservation of this forage (Jian et al., 2017). Ensilage of BB also leads to a reduction in the antinutritive compound (i.e., glucosinolate), improving the nutritional value of the feed (Vipond et al., 1998). Fresh BB is a good source of crude protein (CP; 270 g/kg DM) with relatively low neutral detergent fiber (NDF; 280 g/kg DM) and a metabolizable energy (ME) content of 9.87 MJ/kg DM (Hu et al. 2011) and an in vivo organic matter (OM) digestibility of 86.9% (Wadhwa et al. 2006).

In an in vitro work, the chemical composition, fermentation characteristics, and aerobic stability of cabbage silages either mixed with 40 g ground corn or treated with bacterial inoculant in an attempt to increase DM have been evaluated by Rezende et al. (2015), and it was observed that the inoculant was unnecessary, and the inclusion of ground corn would increase the cost of the silage and, therefore, was impracticable. Previous work on feeding fresh cabbage leaves or cauliflower leaves to goats showed no difference in performance [in terms of apparent digestibility of DM, OM, CP, acid detergent fiber (ADF), efficiency of nutrient utilization, and dry matter intake (DMI)] compared to other green forages (Wadhwa et al., 2006), but it has been illustrated that dietary inclusion of fresh cabbage (up to 200 g/kg diet in concentrate basal feed) to lambs reduced their growth rate (232 vs. 271 g/d) probably due to the presence of S-methylcysteine sulfoxide and glucosinolates, which depress feed consumption (Nkosi et al., 2016). Moreover, lambs fed on Brassica fodder crops, such as broccoli, may develop goiter (broccoli contain goitrogenic compounds, which interfere with the availability of iodine) and this can reduce the lamb performance (Halkier and Gershenzon, 2006). There is a concern over the adverse effects of the profile of glucosinolates when feeding *Brassica oleracea*, as glucosinolates and subsequent metabolites may negatively affect growth performance [European Food Safety Authority (EFSA, 2008)]. Hence, it was hypothesized that BBWS may be fed to Fashandy lambs up to medium concentration (i.e., 200 g/kg of diet DM) without any adverse impacts on health and growth performance.

Therefore, this study was carried out to evaluate the effects of a mixed BB-wheat straw silage (BBWS) substituted only up to 20% of the forage ration (DM basis; i.e., lucerne and wheat straw) in finishing diets for lambs by measuring in vivo apparent digestibility of nutrients, rumen metabolites, N retention, microbial N synthesis (MNS), blood biochemistry parameters, and performance in Fashandy lambs.

## MATERIALS AND METHODS

The Guide for the Care and Use of Agricultural Animals in Research and Teaching (FASS, 2010) was followed for housing, feeding, transport, proper and humane care and use of animals, veterinary care, occupational health and safety, program management and procedures. The Committee of Animal Science of Tarbiat Modares University (Iran) approved the experimental protocols.

### Silage Preparation

Mature BB was harvested according to commercial production practices in Iran (i.e., when the buds of the head are firm and tight before flowering) and chopped into pieces of approximately 5 × 5 cm using an electric cutter (Hallde, RG-200, Sweden). Wheat straw was harvested at full maturity and chopped into 3–5 cm lengths. Broccoli byproduct has a high moisture content (836 g/kg fresh weight), so it was mixed with wheat straw at a ratio of 90:10 on fresh basis (a ratio of 69:31 on a DM basis) to give a fresh mixture containing above 260 g DM/kg of fresh weight, as this DM content promotes relatively well-preserved silage (McDonald et al., 1991). The chopped mixture of BB and wheat straw was ensiled in three trench silos of approximately 1 m height and 5 m width, which were each covered with a thick plastic sheet. The mixture was ensiled and compacted using a tractor, with a compaction density of approximately 900 kg wet matter/m^3^. After ensiling for 60 d, representative samples (500 g) were taken (weekly from five different locations within each silo, which were opened at different time points and stored frozen at −20 °C until analysis) to determine chemical composition and fermentation characteristics.

### Chemical Analysis and Fermentation Characteristics of Silage

Samples of fresh materials and silage were oven-dried at 60 °C to a constant weight and ground to pass through a 1-mm sieve (Wiley mill, Swedesboro, NJ), and the determined DM was corrected for the loss of volatiles during drying. The samples were analyzed for ash (method 924.05), ether extract (EE; method 920.39), and CP (method 988.05) according to AOAC (1998). Ash-free NDF (NDFom) was determined without sodium sulfite (Mertens, 2002). The determination of ADF was performed (method 973.18; AOAC, 1998) and expressed exclusive of residual ash (ADFom). Acid detergent lignin (ADL) was determined by solubilization of cellulose with 72% sulfuric acid (method 973.18), and phenolic compounds using the Folin–Ciocalteu method. The water-soluble carbohydrates (WSC) were measured by colorimetry after reaction with anthrone reagent (MAFF, 1982). The ME contents of pre-ensiled and BBWS were calculated according to Menke et al. (1979) as:

$$\begin{array}{ll} ME\;\left(MJ/kg\;DM\right)= & \;2.20\;+0.136\times GP_{24}\\ & +0.057\times CP+0.0029\times EE^{2} \end{array}$$

where GP_24_ is 24-h net gas production (mL/200 mg of DM), and CP is measured in g/kg of DM.

For measuring silage pH, 50 g of fresh silage was blended with 125 mL of distilled water and allowed to stand at room temperature for 1 h (Faithfull, 2002). After decanting the silage extract into a small beaker, the pH was measured using a portable digital pH meter (Sartorius PT-10; Sartorius AG, Göttingen, Germany). To determine ammonia-N (NH_3_-N), an extract was obtained by squeezing the silage material, filtered using Whatman 54 filter paper, then a 9 mL of aliquot was taken, mixed with 1 mL of 7.2 N H_2_SO_4_, and stored at −20 °C. After thawing, the silage extracts were analyzed for NH_3_-N using a phenol-hypochlorite assay (Galyean, 1997). Two milliliters of the silage juice was pipetted into a microcentrifuge tube with 0.5 mL of an acid solution (containing 20% orthophosphoric acid and 20 mM 2-ethyl butyric acid, as the internal standard) and centrifuged at 15,000 × *g* for 15 min at 4 °C (Rezaei et al., 2014). The supernatant was used to quantify the lactic acid and volatile fatty acids (VFA) using a gas chromatograph (UNICAM 4600; SB Analytical, Cambridge, UK) equipped with a flame ionization detector (250 °C), split-injection port (1.0 μL injection), and a capillary column (Agilent J&W HP-FFAP, 10 m by 0.535 mm by 1.00 μm, 19095F-121; Agilent, Santa Clara, CA). The carrier gas was He (column head pressure of 68.9 kPa) and the working temperature of the injector and detector were 250 and 300 °C, respectively. The initial column temperature was set at 80 °C for 1 min and then increased with 20 °C/min to 120 °C followed by 6.2 °C/min to 140 °C and thereafter with 20 °C/min to 205 °C.

### Treatments, Animals, and Diets

Forty-five male Fashandy lambs with an average BW of 29.9 ± 0.9 kg and 6 months of age were used in this study. Animals were housed in individual wooden-slatted pens (1.5 × 1.5 m) and given an adaptation period of 14 d followed by a data collection period of 56 d. Lambs were offered isonitrogenous and isocaloric diets, which were daily (Table 1) formulated according to National Research Council (NRC, 1985) guidelines, in which forage (lucerne + wheat straw) was replaced with different concentrations (0, 100, or 200 g/kg of diet DM) of BBWS (Table 2). Diets were randomly assigned by BW into three groups of 15 lambs each in a completely randomized design. Animals received the diets as a total mixed ration, at 0800 and 1700 h, to ensure 10% feed refusal and had constant access to freshwater.

**Table 1.** Ingredients and chemical composition (% of DM) of the diets containing different concentrations of BBWS

	Concentration of BBWS in diet (% of DM)		
Item	0	10	20
Ingredient			
Alfalfa hay	15	12	7.5
Wheat straw	12.5	5.5	—
Wheat bran	19.6	19.6	19.6
BBWS	—	10.0	20.0
Corn grain	44.3	44.3	44.3
Soybean meal	5.9	5.9	5.9
Urea	0.5	0.5	0.5
Sodium bicarbonate	0.7	0.7	0.7
Calcium carbonate	0.7	0.7	0.7
Vitamin–mineral premix*	0.5	0.5	0.5
Salt	0.3	0.3	0.3
Chemical composition†			
DM			
OM	94.7	95.1	93.3
CP	14.6	14.8	14.5
NDFom	28.3	27.1	25.8
EE	7.0	7.9	7.9
ME, Mcal/kg of DM	2.69	2.69	2.67

*Contained (per kilogram) 190 g of Ca, 50 g of Na, 19 g of Mg, 3 g of Zn, 3 g of Fe, 2 g of Mn, 300 mg of Cu, 100 mg of Co, 1 mg of Se, 100 mg of I, 100 mg of vitamin E, 500,000 IU of vitamin A, 100,000 IU of vitamin D3, 300 mg of antioxidant. †Calculated from analysis of each feed composition, which were analyzed.

**Table 2.** Chemical composition (% of DM), fermentation characteristics, and ME values (Mcal/kg of DM) of pre-ensiled and ensiled BBWS

Item	Pre-ensiled	Ensiled	SEM	*P*-value
Chemical composition				
DM, % of fresh weight	26	30	1.4	0.003
CP	9.0	8.0	0.46	0.05
NDFom	39	40	0.97	0.16
ADFom	32.6	36.4	1.3	0.03
Acid detergent lignin	6.5	11.2	1.7	0.001
Ash	18	27	0.36	0.001
EE	5.9	7.4	0.16	0.02
Water-soluble carbohydrate	7.5	0.5	0.08	0.0001
ME, Mcal/kg of DM	1.91	1.72	0.02	0.015
TP*	0.6	0.55	0.08	0.16
Fermentation characteristics				
pH value	—	4.4		—
Ammonia-N, % of total N	—	6.1		—
Fermentative acids, % of DM				
Lactic acid	—	4.5		—
Acetic acid	—	1.7		—
Propionic acid	—	0.2		—
Butyric acid	—	0.5		—
Valeric acid	—	0.04		—
Isovaleric acid	—	0.05		—

*Total phenolic compounds.

### Feed Consumption and Growth Performance

The feed eaten by each animal and corresponding feed refusals were recorded daily to estimate the consumption of DM and nutrients, and representative samples were kept frozen at –20 °C for subsequent analyses. Samples of feed offered and refusals were oven-dried at 60 °C to a constant weight, ground to pass through a 1-mm sieve, then analyzed for OM, N, and EE as described above. The silos of BB-wheat straw, which were opened at different time points (i.e., on days 1, 21, 41, and 61 of the trial), were fed to animals. The body weight (BW) of animals was individually recorded at the beginning and the end of the experiment period (on two consecutive days to provide baseline weights) and at 15-d intervals before the morning feeding, after 16 h fasting, to calculate average daily gain (ADG) and feed efficiency (FE). The ADG was calculated for each lamb by using the following equation:

$$\begin{array}{ll} ADG= & \;\left(f\;inal\;BW\;-\;initial\;BW\right)/\\ & \;number\;days\;on\;feeding. \end{array}$$

### In Vivo Apparent Digestibility of Nutrients, Microbial N Supply, and N Retention

On day 56 of the data collection period, randomly four animals per treatment were placed into individual metabolism crates (0.6 × 1.3 m), allowing feces and urine to be collected. Apparent digestibility was determined by the total fecal collection method, with 6 d for sample collection. During the collection period, the amounts of feed offered, feed refusals, and feces from each animal were recorded daily and samples were stored (at –20 °C) for later analysis of DM, OM, CP, NDFom, and EE as described above.

Total urine produced daily was collected in plastic vessels containing 100 mL of 10% sulfuric acid. From the daily urine collected, 10% was sampled, diluted fivefold with distilled water, and then stored at –20 °C to estimate purine derivatives and nitrogen (Chen and Gomes, 1992). Urine allantoin was measured by a colorimetric method at 522 nm after its conversion to phenylhydrazine. The sum of xanthine and hypoxanthine was calculated by their conversion to uric acid with xanthine oxidase (X-1875; Sigma-Aldrich, St. Louis, MO) and measuring the subsequent optical density at 293 nm. Uric acid was measured from the reduction in optical density at 293 nm after the degradation of uric acid to allantoin with uricase (U-9375; Sigma-Aldrich). Total purine derivative excretion per day was calculated, the daily absorbed exogenous purines were estimated, and the MNS was predicted. The concentration of urinary N was estimated by the Kjeldahl method (AOAC 1998), and N retention was calculated as daily N excretion (urinary N plus fecal N) subtracted from daily N intake.

### Rumen Metabolites

Just before the morning feeding and 2, 4, and 6 h afterward, rumen fluid from four animals randomly allocated to each treatment was sampled (using an esophageal tube) on day 56. After discarding the first 15 mL of rumen fluid, pH was determined. For NH_3_-N determination, a 25-mL sample of strained rumen fluid was preserved with 5 mL of 0.2 N HCl at –20 °C and then analyzed using a phenol-hypochlorite assay (Broderick and Kang, 1980). To measure ruminal VFA, 8 mL of strained rumen fluid was preserved (at –20 °C) with 2 mL of an acid solution (containing 20% orthophosphoric acid and 20 mM 2-ethyl-butyric acid as internal standard). After thawing, rumen fluid was centrifuged (15,000 × *g* for 15 min at 4 °C) and the supernatant was used to quantify VFA as described previously.

Rumen protozoa were enumerated using 5 mL of rumen fluid pipetted into a test tube containing 20 mL of formalinized physiological saline (20 mL of formaldehyde in 100 mL of saline containing 0.85 g of NaCl in 100 mL of distilled water). Total and subfamily counts of protozoa were determined using a light microscope and a Hemocytometer (Neubauer-improved; MarienfeldSuperior, Lauda-Königshofen, Germany; Dehority 2003).

### Blood Biochemistry Parameters

Just before the morning feeding and 4 h after feeding, blood samples (15 mL) were randomly collected from four animals assigned to each treatment on days 56 and 70 (the same animals were used on both times). The samples were transferred to the laboratory on ice, and the harvested serum was stored at –20 °C until analysis. Glucose, triglyceride, cholesterol, urea N, total protein, albumin, and creatinine concentrations were measured using kits from Pars Azmun (Tehran, Iran) using a spectrophotometer (Genova; Jenway, Essex, UK). Blood thyroxine and triiodothyronine concentrations were measured using kits from Pars Azmun (Tehran, Iran) using an ELISA reader.

### Statistical Analysis

Using a *t*-test, the chemical composition of pre-ensiled and BBWS were compared to quantify changes made during the process of silage fermentation (Table 2). Data on ADG, digestibility, MNS, and N retention were statistically examined using the PROC MIXED of SAS (Version 9.1; SAS Inst. Inc.) in a completely randomized design. The model used was

$$\begin{array}{l} y_{ijk}=\mu +T_{i}+e_{ij}+e_{ijk}, \end{array}$$

where *y*_ij_ represents the value of each individual observation, µ is the overall mean, *T*_*i*_ is the fixed effect (treatment) of the *i*th concentration of BBWS (*i* = three concentrations of BBWS), *e*_ij_ represents the experimental error, and *e*_ijk_ represents the sampling error. The random effect included treatment × replication. The model included the fixed effect of dietary treatment. Moreover, data on digestibility, MNS, and N balance were measured once and examined using the same model with four replicates for each diet.

Repeated measures were used to statistically examine the data of rumen metabolites and blood biochemistry parameters. The model used was

$$\begin{array}{l} y_{ijk}=\mu +\;T_{i}+\;\sigma_{ij}+\;S_{k}+\;T_{i}S_{k}+{\;\varepsilon \;}_{ijk} \end{array}$$

where *y*_ijk_ represents the value of each individual observation, µ is the overall mean, *T*_*i*_ is the fixed effect (treatment) of the *i*th concentration of BBWS (*i* = three concentrations of BBWS), σ _ij_ is the main random error, *S*_*k*_ is the sampling time, T_i_S_k_ is detecting interaction between treatment and sampling time, and Ɛ _ijk_ is the subrandom error. The influence of diet (treatment), sampling time, and their interactions were considered fixed. Compound symmetry was used based on the lowest Akaike information criterion (AIC) as a covariance structure in the model. Also, repeated measure was used to test the data on feed consumption, whereas the influence of dietary treatment, and their interactions were considered fixed.

Additionally, a polynomial contrast was used to test the linear or quadratic effects of the diet (i.e., BB inclusion concentration) on parameters measured. The result is then illustrated as statistically significant at *P* ≤ 0.05.

The SAS program was used to calculate the number of replications needed to obtain the desired power (>0.8). The power of the test is given by:

$$\begin{array}{l} Power\;=\;P\;\left(F\;>\;F_{\alpha ,\left(a-1\right),(N-a)}=\;F_{\beta }\right) \end{array}$$

using a noncentral *F* distribution for *H*_1_ with a noncentrality parameter λ = SS_treatment_/MS_error_, and degrees of freedom (*a* − 1) and (*N* − *a*). Here, *N* is the number of replications per treatment, *a* is the number of treatments, and *F*_*α,*(*a −* 1),(*N −* a)_ is the critical value (Kaps and Lamberson 2009).

## RESULTS

### Chemical Analysis and Fermentation Characteristics of Silage

Compared to the fresh product (Table 2), BBWS contained higher concentrations of ADFom (*P* < 0.03), ADL and ash (*P* < 0.001), and lower WSC concentration (*P* < 0.0001).

### In Vivo Apparent Digestibility of Nutrients, Feed Intake, and Growth Performance

The apparent digestibility of DM, OM, CP, NDFom, and EE was not influenced (*P* < 0.34, *P* < 0.39, *P* < 0.89, *P* < 0.69, and *P* < 0.35, respectively) by feeding BBWS (Table 3). The DMI, organic matter intake, crude protein intake, ether extract intake, neutral detergent fiber intake, metabolisable energy intake, ADG, and feed conversion ration (FCR) were not affected (*P* < 0.67, *P* < 0.67, *P* < 0.66, *P* < 0.30, *P* < 0.63, *P* < 0.32, *P* < 0.55, and *P* < 0.55, respectively) by feeding the experimental diets.

**Table 3.** Effect of dietary inclusion of BBWS on in vivo apparent digestibility (*n* = 4), intake, and growth performance of fattening lambs

	Concentration of BBWS in diet (g/kg of DM)				
Item	0	100	200	SEM	*P*-value
Digestibility, %					
DM	76	75	73	1.50	0.34
OM	77.1	76	78.1	0.75	0.39
CP	72.1	72.5	72.4	2.4	0.89
NDFom	62	61	63	2.85	0.69
EE	77	77	79	1.34	0.35
Intake, g/d					
DM	1,432	1,435	1,420	14.4	0.67
OM	1,332	1,316	1,315	16.25	0.67
CP	204	200	202	2.59	0.66
EE	112	113	115	1.77	0.3
NDFom	420	414	416	4.67	0.63
ME, Mcal/d	2.94	2.91	2.96	0.03	0.32
Performance					
Initial BW	29.41	29.28	29.06	0.87	0.28
Finial BW	42.8	42.47	41.94	1.20	0.32
ADG, g/d	239	235	230	9.34	0.55
FCR*	5.99	6.12	6.13	0.4	0.55

*Feed conversion ratio (DMI:ADG).

### Microbial N supply and N retention

Feeding lambs with BBWS had no effect (*P* < 0.53) on total purine derivatives absorbed, total purine derivative excretion (*P* < 0.56), and MNS (*P* < 0.57; Table 4). Among the lambs, there were no significant changes in N consumption (*P* < 0.72), fecal N (*P* < 0.88), urinary N (*P* < 0.61), and N retained (*P* < 0.81).

**Table 4.** Effect of dietary inclusion of BBWS on total purine derivatives (TPD), MNS, and N retention in fattening lambs (*n* = 4)

	Concentration of BBWS in diet (g/kg of DM)				
Item	0	100	200	SEM	*P*-value
Urinary excretion, mmol/d					
Allantoin	8.58	8.04	8.10	0.56	0.41
Xanthine + hypoxanthine	0.73	0.73	0.74	0.51	0.51
Uric acid	3.61	4.10	4.37	0.55	0.52
TPD absorbed, mmol/d	14.69	14.67	15.11	1.07	0.53
TPD excretion, mmol/d	12.92	12.87	13.22	0.91	0.56
MNS, g/d	10.68	10.66	10.99	0.78	0.57
N retention, g/d					
N intake	29.7	29.02	29.12	1.10	0.72
Fecal N	8.17	8	8.01	0.69	0.88
Urinary N	11.27	11.07	11.10	0.50	0.61
Retained N	10.28	9.95	10.01	0.65	0.81
Retained N, g/kg of N intake	346	343	344	2.81	0.96

### Rumen Metabolites and Protozoa Enumeration

Dietary inclusion of BBWS had no effect on ruminal pH (*P* < 0.82) and NH_3_-N concentration (*P* < 0.61; Table 5). Feeding BBWS had no influence on the content of total VFA (*P* < 0.83), acetic (*P* < 0.49), propionic (*P* < 0.63), or butyric acid (*P* < 0.41) concentrations. Ruminal enumeration of total protozoa, *Entodiniinae*, *Diplodiniinae*, *Isotrichidae*, *Epidiniinae*, and *Ophrioscolecinae* subfamilies were not affected by feeding lambs with different concentration of BBWS (*P* < 0.55, *P* < 0.78, *P* < 0.57, *P* < 0.17, *P* < 0.65, and *P* < 0.29, respectively).

**Table 5.** Effect in vivo of dietary inclusion of BBWS on rumen metabolites and protozoa numeration in fattening lambs (*n* = 4)

	Concentration of BBWS in diet (g/kg of DM)				
Item	0	100	200	SEM	*P*-value
pH	6.38	6.37	6.43	0.14	0.82
Ammonia-N, mg/dL	10.12	10.33	10.50	0.86	0.61
Total VFA, mmol/L	82.3	79.63	81.95	1.7	0.83
VFA, mmol/100 mmol					
Acetic acid	56.29	54.53	54	1.66	0.49
Propionic acid	27.11	28.5	28.58	1.60	0.63
Butyric acid	15.9	16.3	16.8	0.44	0.41
Valeric acid	0.56	0.55	0.50	0.08	0.76
Isovaleric acid	0.14	0.12	0.12	0.02	0.63
Acetate:propionate	2.08	2.08	2.06	0.22	0.94
Protozoa, log_10_/g of digesta					
Total protozoa	6.09	6.06	6.06	0.032	0.55
*Entodiinina*	5.86	5.83	5.86	0.039	0.78
*Diplodiinina*	5.45	5.40	5.35	0.086	0.57
*Isotrichidea*	4.87	4.86	4.98	0.042	0.17
*Epidiniina*	4.79	4.78	4.79	0.013	0.65
*Ophrioscolecinae*	4.89	5.01	5.04	0.06	0.29

No significant interaction between treatment and sampling time were observed.

### Blood Biochemistry Parameters

Table 6 illustrated that the blood concentrations of glucose, triglyceride, cholesterol, urea N, creatinine, triiodothyronine, thyroxin, total protein, albumin, and globulin, did not change (*P* < 0.59, *P* < 0.85, *P* < 0.75, *P* < 0.52, *P* < 0.63, *P* < 0.59, *P* < 0.76, *P* < 0.72, *P* < 0.68, and *P* < 0.75, respectively).

**Table 6.** Effect of dietary inclusion of BBWS on blood biochemistry parameters in fattening lambs

	Concentration of BBWS in diet (g/kg of DM)				
Item*	0	100	200	SEM	*P*-value
Metabolites, mg/dL					
Glucose	67.22	69.41	70.76	1.52	0.59
Triglyceride	21.98	21.23	21.90	2.10	0.85
Cholesterol	59.60	61.70	63.99	5.38	0.75
Blood urea N	19.45	22.44	19.58	1.27	0.52
Creatinine	1.40	1.42	1.45	0.07	0.63
Thyroid hormones, ng/mL					
Triiodothyronine	5.13	5.25	5.33	0.28	0.59
Thyroxin	69.00	70.76	71.80	3.30	0.76
Protein, g/dL					
Total protein	6.77	6.67	6.76	0.18	0.72
Albumin	3.59	3.51	3.55	0.11	0.68
Globulin	3.18	3.16	3.20	0.15	0.75
Albumin:globulin	1.14	1.10	1.11	0.06	0.68

*Average of repeated sampling of blood collected from six animals assigned to each treatment on days 56 and 70 of data collection period, just before the morning feeding and 4 h after the morning feeding.

## DISCUSSION

### Chemical Analysis and Fermentation Characteristics of Silage

Changes in the chemical composition of BBWS after ensilage occur because of microbial fermentation and losses from effluent throughout the ensiling process (Buxton and O’Kiely, 2003), but the DM of BBWS is within the range of medium- to good-quality silage. Low-moisture silage depresses undesirable clostridial growth (Pahlow et al., 2003) because of the affinity of *Clostridium* spp. for moisture. The production of butyric acid was low in the present study, indicating low growth of *Clostridium* spp., which derive energy for growth by fermenting sugars and lactate into butyric acid (Pahlow et al., 2003). In agreement with our results, Shao et al. (2007) documented improved fermentation of perennial ryegrass with decreased concentrations of butyric. The inverse relationship between ADFom, ADL, and WSC show that a reduction in WSC increases the content of ADFom and ADL resulting in a proportional rise in ash concentration (Megías et al., 2014). The preservation of BBWS was suitable in terms of pH value (i.e., 4.8), low NH_3_-N content (<100 g/kg total N), and low concentrations of acetic, propionic, and butyric acids (McDonald et al., 2011). A good-quality silage is characterized by a lactic acid concentration between 30 and 140 g/kg of DM (McDonald et al., 2011), and BBWS lactic acid concentration was 45 g/kg of DM. The pH values of our BBWS were the same as those (pH = 4.76) noted by Jian et al. (2017) who ensiled rice straw, broccoli residue, and lucerne at a ratio of 5:4:1 for 30 d, whereas, for other silage characteristics, such as NH_3_-N, lactic acid, and VFA content, Jian et al. (2017) reported a lower silage quality than those observed in BBWS.

### In Vivo Apparent Digestibility of Nutrients, Feed Consumption, and Growth Performance

The inclusion of BBWS in the diets had no effect on nutrient digestibility and this relates to the similar chemical composition of the diets and the comparable ruminal pH in the lambs, factors which influence rumen microbial activity (Petit and Castonguay, 1994). The low concentration of phenolic compounds (1.0 g/kg of DM) had no adverse effect on diet apparent digestibility. Previous work on fresh cauliflower (*Brassica oleracea* var. *botrytis*) reported good nutrient digestibility of DM, OM, CP, and NDF (80.9%, 86.9%, 84.9%, and 71.8%, respectively) when fed to goats (Wadhwa et al., 2006). It has been stated that increased concentrations of fresh cabbage (*Brassica oleracea* var. *capitata*) in the diet of lambs reduced OM and NDF digestibility (73% vs. 65%; 56% vs. 47%, respectively; Nkosi et al., 2016).

 Feeding BBWS had no influence on feed consumption and this may be due to the similar diet digestibility, particularly NDFom digestibility. It was noted that NDF digestibility provides an accurate estimation of intake (Harper and McNeill, 2015). A correlation between NDF digestibility and intake was stated by Nkosi et al. (2016) who noted a lower DMI for lambs fed fresh Brassica (*oleracea var. capitata*) compared to a Brassica-free diet (1,420 vs. 1,600 g).

The similar ADG among the lambs reflected their comparable ruminal VFA concentration and MNS (Ben Salem et al., 2002). The FCR showed no difference among the lambs due to their similar DMI and ADG. The experimental diets, in the current study, were formulated to meet the requirements of a growth rate of 300 g/d for fattening male lambs at 8.5 mo of age according toNRC (1985) recommendations. However, the ADG of our lambs was not as predicted in NRC (1985) tables because the equations used to predict lamb growth were not obtained in Iranian sheep. Iranian sheep breeds (except the Zel breed) are fat tailed and their physiological characteristics and genetic potential for growth and, to some extent, the rumen microbiota of these breeds differ from sheep in other countries, which can lead to a difference in overall performance.

### Microbial N Supply and N Retention

Diets that maximize MNS often increase animal performance (Chen and Gomes, 1992). In the present study, the MNS reached its highest value (10.99 g/d) with 200 g of BBWS per kilogram of diet DM. Similar concentrations of urinary purine derivatives (PD) and MNS among treatments were due to the similar intakes of digestible OM and N (Ben Salem et al., 2002) and the equal ruminal synchrony of ME and N resulting from a similar daily ME intake and digestible CP consumption among the treatments. Urinary PD excretions are indicators of MNS arriving at the beginning of the small intestine (Chen and Gomes, 1992). It was reported by Wadhwa et al. (2006) that higher MNS values in goats offered fresh cauliflower (*Brassica oleracea* var. *botrytis*; 25.40 g/d) compared to those in the current study. This difference could be due to the different experimental animals and diets (i.e., lambs vs. goats; BBWS vs. fresh *Brassica oleracea*).

Nitrogen retention among the animals did not differ because digestible OM and N (Ben Salem et al., 2002), N excreted in feces and urine (Zuo, 2011), and ADG were not influenced by feeding BBWS. A lower N consumption and N retention were noted in animals offered diets with cabbage compared to those fed a Brassica-free diet, suggesting that the lower N intake and retention could be related to the decrease in the digestibility of DM and OM in this diet (Nkosi et al., 2016).

### Rumen Metabolites and Protozoa Enumeration

For all lambs, ruminal pH values varied from 6.37 to 6.43, which were within the optimum range (i.e., 6.1–6.6) for maintaining normal cellulolytic organism populations (Van Soest, 1994). Feeding BBWS had no effect on rumen pH and this was related to the similar DMI and nutrient digestibility among lambs in different treatments. Dietary addition of BBWS had no adverse effects on ruminal metabolism. It was noted by Keogh et al. (2009) that replacing grass silage with fresh kale (*Brassica oleracea*) had no effect on the ruminal pH of dry dairy cows (mean pH = 6.2). In contrast, feeding fresh brassica forage (*Brassica spp*.) to lambs resulted in a lower ruminal pH (<6.0) as a result of the greater ratio of readily fermentable to structural carbohydrates in *Brassica* forage (Sun et al., 2015). Ruminal NH_3_-N content in all the animals was higher than 5 mg/dL, which is the minimum concentration required by rumen microorganisms to support their optimum growth (McDonald et al., 2011). The lack of difference in rumen NH_3_-N concentration among the lambs suggests that the release of N in the rumen and its uptake by microbes to synthesize microbial CP were not affected by the diets.

Ruminal VFA is the main energy source for ruminants (Abarghuei et al., 2013); consequently, diets that increase the ruminal production of VFA lead to higher animal performance (Penner, 2014). In the current study, dietary BBWS had no influence on the total and proportions of individual VFA content; thus, the performance of fattening lambs was not affected by the diets. The similar daily OM intake and OM digestibility, which resulted in equal fermented OM per day, reflects the similar ruminal concentrations of total and proportions of individual VFA among the dietary treatments (Morvay et al., 2011).

Rumen protozoa, exclusively ciliates, rank second only to bacteria in cellular biomass of the ruminal microbiota (Dehority, 2003). They are found in the rumen where they play important roles in feed digestion and homeostasis of the rumen ecosystem (Dehority, 2003). Feeding BBWS had no effect on rumen protozoa enumeration and this was due to the normal ruminal pH, which plays an important role in rumen protozoa activity (Dehority, 2003).

### Blood Biochemistry Parameters

Concentrations of blood parameters represent an integrated index of the adequacy of nutrient supply in relation to the utilization of nutrients, which is a self-governing aspect of the physiological condition of the animal and provides an immediate indication of nutritional status at a specific point in time (Pambu-Gollah et al., 2000). In the present study, the lack of differences in blood biochemistry parameters among the treatments was probably due to similar intakes and nutrient digestibilities. The serum concentrations of glucose, cholesterol, urea N, total protein, albumin, and globulin and the albumin to globulin ratio in all treatments were within the normal ranges for sheep (Radostits et al., 2007). However, the concentration of triglyceride was greater than the range for sheep (Radostits et al., 2007) but was in the usual range reported for fat-tailed Iranian sheep (18.03 to 50.93 mg/dL; Mojabi, 2011). The similar concentrations of blood albumin among the lambs suggested normal liver function as the liver is the main organ of albumin synthesis. The values of albumin and globulin in the serum of the lambs in the current study show that these animals did not suffer from any health problems that might have affected their performance (Radostits et al., 2007).

In Brassicas, the antinutritional S-methylcysteine sulfoxide and glucosinolates were reported to be goitrogenic and could affect triiodothyronine and thyroxin production (Chorfi et al., 2015). The lambs offered BBWS had no clinical indicators of glucosinolate toxicity because the concentration was low and there were no symptoms of toxicity (EFSA, 2008). This may be due to the ensiling process causing a reduction in the concentration of glucosinolates and S-methylcysteine sulfoxide improving the feed quality (Vipond et al., 1998). Similarly, feeding different concentrations of ensiled kale (*Brassica oleracea*) in lambs did not affect their performance or blood triiodothyronine and thyroxin concentrations probably because of the reduction in glucosinolate during ensilage (Vipond et al., 1998).

## CONCLUSIONS

The chemical composition and fermentation characteristics of BBWS indicates its potential as a ruminant feedstuff. BBWS inclusion at up to 200 g/kg of DM in the diet of fattening Fashandy lambs is equivalent to an Alfalfa-wheat straw diet in terms of effects on nutrient digestibility, feed intake, growth performance, FE, rumen parameters and blood parameters. BBWS at this level is a safe feedstuff for sheep having no adverse effects on their health status or growth performance.

*Conflict of interest statement.* None declared.
